# Different Expression Pattern of TIM-3 and Galectin-9 Molecules by Peripheral and Peritoneal Lymphocytes in Women with and without Endometriosis

**DOI:** 10.3390/ijms21072343

**Published:** 2020-03-28

**Authors:** Matyas Meggyes, Laszlo Szereday, Noemi Bohonyi, Miklos Koppan, Sarolta Szegedi, Anna Marics-Kutas, Mirjam Marton, Anett Totsimon, Beata Polgar

**Affiliations:** 1 Department of Medical Microbiology and Immunology, Clinical Centre, Medical School, University of Pecs, 12 Szigeti Street, 7624 Pecs, Hungary; meggyes.matyas@pte.hu (M.M.); szereday.laszlo@pte.hu (L.S.); totsimonanett98@gmail.com (A.T.); 2 Janos Szentagothai Research Centre, University of Pecs, 20 Ifjusag Street, H-7624 Pecs, Hungary; 3 Department of Obstetrics and Gynecology, Clinical Centre, Medical School, University of Pecs, 17 Edesanyak Street, H-7624 Pecs, Hungary; noemibohonyi@gmail.com (N.B.); koppan.miklos@pte.hu (M.K.); szegedisarolta@gmail.com (S.S.); kutas.anna.md@gmail.com (A.M.-K.)

**Keywords:** endometriosis, TIM-3, Galectin-9, flow cytometry, immunology, pathogenesis

## Abstract

Endometriosis is a gynecological condition that is associated with chronic pelvic inflammation, pain, and infertility. Although substantial evidence supports that immunological alterations contribute to its pathogenesis and we previously posed a pivotal role of Galectin-9 (Gal-9) in this disorder, the involvement of the TIM-3/Gal-9 pathway in the development of endometriosis-associated immunological abnormalities is not yet known. In the present study, multicolor flow cytometry was used to compare the immunophenotype and cell surface expression of TIM-3 and Gal-9 molecules on peripheral blood (PB) and peritoneal fluid (PF) lymphocytes of women with and without endometriosis. We found an altered distribution of different lymphocyte subpopulations, a markedly decreased TIM-3 labeling on all T and NK subsets and a significantly increased Gal-9 positivity on peripheral CD4+ T and Treg cells of the affected cohort. Furthermore, a significantly increased TIM-3 expression on CD4+T-cells and elevated Gal-9 labeling on all T and NK subsets was also revealed in the PF of the examined patients. In conclusion, our results suggest a persistent activation and disturbed TIM-3/Gal-9-dependent regulatory function in endometriosis, which may be involved in the impaired immune surveillance mechanisms, promotes the survival of ectopic lesions, and aids the evolution of reproductive failures in endometriosis.

## 1. Introduction

Endometriosis is a common chronic, progressive gynecologic condition that influences the quality of life and can lead to infertility. This complex, estrogen-dependent neuroimmunoendocrine disorder affects 3–10% of women in their reproductive years and 20–50% of women with infertility [1]. It is defined by the presence of endometrial glandular cells; stromal cells are growing and developing outside the uterine cavity, mainly on the pelvic peritoneum and/or ovaries. Typical symptoms of endometriosis are not pathognomonic and include menstruation-associated chronic pelvic pain, dyspareunia, dysmenorrhea, pelvic inflammation, and infertility [2]. Currently, its medical treatment is mainly based on surgery and/or ovarian suppressive agents that could be effective in the temporary relief of symptoms and infertility. Still, up to this day, no treatment is available to cure the disease [3].

It is well known that multiple factors are responsible for the aetiopathogenesis of endometriosis involving a variety of environmental, genetic, epigenetic, endocrine, or immune factors [4,5]. While the pathophysiology of the disease is still unknown, there is growing evidence that endometriosis is associated with disturbed local and systemic immune responses [6]. Impaired cellular and humoral immunity, increased activity of peritoneal macrophages, altered function of neutrophils, dendritic cells, T and B lymphocytes, and decreased natural killer (NK) cell activity are all involved in its immunopathogenesis [7]. In addition, an elevated level of Forkhead box P3 (FoxP3) expressing regulatory T cells (Treg) [8] and altered production of growth factors and cytokines could also play a crucial role in the implantation of ectopic lesions [9]. Further studies have found a link between endometriosis and oxidative stress [10] or microbial dysbiosis [11]. 

Originally described as a strong eosinophil chemoattractant, Galectin-9 (Gal-9) is one of the members of the tandem-repeat type galectin family containing two carbohydrate recognition domains and a linker peptide with an affinity for β-galactosides [12]. Gal-9 has several isoforms [13,14,15] and is encoded on the short arm of chromosome 17. The LGALS9 gene consists of 11 exons whose alterative splicing can influence the valency and function of the encoded molecules [16]. Many cell types expressing Gal-9 [17] considered a known pleiotropic immune-modulator either by ameliorating the inflammatory process [18] or by exhibiting anti-inflammatory properties. It can activate inflammatory response and enhance the production of pro-inflammatory cytokines by monocytes and T helper 1 (Th1) cells. In addition, it is capable of inducing the apoptosis of Th1, Th17, and to a lesser extent, CD8+ T cytotoxic effector cells promote the differentiation of naïve T cells into Tregs by increasing FoxP3 expression [12] and suppress the differentiation of Th17 lymphocytes [19]. A recent study identified a novel CD4+ Th cell subset that expresses Gal-9 on its surface and secretes Gal-9 upon T cell receptor (TCR) stimulation, thereby regulate Th17/Treg development [20]. The therapeutic potential of Gal-9 is proved in several autoimmune disease models [21], in transplantation [22], in allergic asthma [23], and different malignancies [12]. 

Gal-9 can interact with various extracellular matrix proteins and cell surface ligands, as well as the inhibitory T-cell immunoglobulin and mucin domain-3 (TIM-3) receptor. The TIM-receptor family consists of three members in humans (TIM-1,-3,-4) and eight in mice (TIM-1-8). They are expressed by various immune cells, including T, NK, and antigen-presenting cells, and implicated in the regulation of pivotal immunological processes such as T cell proliferation, survival, and tissue inflammation [24,25]. The amount of data supports that TIM-3 is a potent regulator of both the adaptive and innate immune response, and negatively regulates T cell responses by inducing the apoptosis of Th1 cells [26]. To date, numerous studies investigated the interaction between Gal-9 and TIM-3 and found that the commitment of TIM-3 by Gal-9 may function as a regulator of Th1 immunity by abrogating Th1/Th17-driven immune responses, therefore modulate the Th1/Th2 cytokine balance [20]. A growing body of evidence demonstrates that galectins are widely expressed in the female reproductive system. Although it is well established that these lectins can control host-pathogen interactions, the function of the endometrium, the local immune response as well as implantation, placentation, and immune tolerance towards the fetus [27], their exact role in endometriosis is poorly examined. Previous literature data indicate that Gal-9 is mainly expressed by endometrial epithelial cells [28], and its production can be induced by tissue damage or pro-inflammatory cytokines (IFNγ, IL-1β, TNFα [29,30]). Furthermore, we found an overexpression and an elevated serum level of Gal-9 in endometriosis [31]. In this regard, we hypothesize, that the dysregulated TIM-3/Gal-9 immune checkpoint pathway has a crucial role in the immuno-pathogenesis of this disease. 

Here we demonstrate that the distribution and the cell surface expression of the TIM-3 receptor and the Gal-9 ligand are significantly altered on different peripheral and peritoneal T and NK cell subsets of endometriosis-affected patients. Furthermore, our results indicate a persistent activation and a dysregulated TIM-3-dependent regulatory pathway in endometriosis, which may impair the local immune-surveillance mechanisms, could promote the survival of ectopic endometriotic implants and aid the development of endometriosis-associated reproductive failures.

## 2. Results

### 2.1. Immunophenotypic Characterization of Peripheral Blood and Peritoneal Fluid Mononuclear Cells in Patients with Endometriosis and Non-endometriotic Women

As the exact immunophenotypic composition of the peripheral blood (PB) and peritoneal fluid (PF) immune cell populations in endometriosis were not yet characterized by our Research Team and previous literature data provided inconsistent results, our first aim was to determine the distribution of different T and NK cells in the peripheral blood mononuclear cell (PBMC) subsets of non-endometriotic (NE) control and endometriosis (E)-affected women and in the peritoneal fluid leukocytes (PTL) of the examined patients. Although all E-cases were confirmed by laparoscopy and their demographic data was evaluated in detail (Table 1), but the clinical classification was not done as the patients suffered from different forms and various extents of the disease. Unfortunately currently, neither the best-known rASRM nor the ENZIAN scoring system could accurately be used for the unified, comparative classification of all lesion types [32,33,34]. Moreover, the parallel use of both scoring systems for classification would be inappropriate as they refer to the different locations of lesions. Although the revised ENZIAN scoring may provide an excellent complement to the rASRM score for a morphological description of deep infiltrating endometriosis (DIE), however, this system is not in use in our clinical practice [35]. In the case of NE-cohort, under the EU-General Data Protection Regulation (EU-GDPR) and the Privacy, Informational, and Healthy data Act, with special regard to the confidentiality and privacy of voluntary non-remunerated blood donation, all personal and health-related data obtained about the donors during blood donation were processed anonymously, confidentially and securely and were not disclosed to the Research team for analysis.

**Figure 1:**
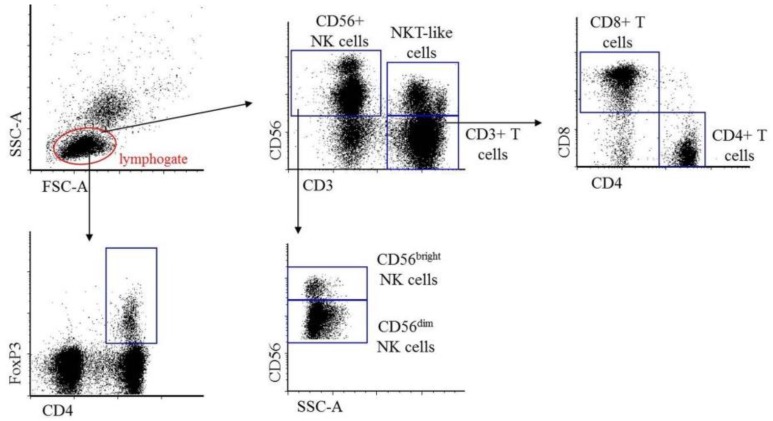
Gating strategy for flow cytometry analysis. Selection method of the investigated peripheral and peritoneal CD3+ T, CD4+ T, CD8+ T, CD4+ Treg, CD56+ NK, CD56^dim^ NK, CD56^bright^ NK, and NKT-like immune cell subpopulations.

During analysis, the percentage of CD3+ T, CD4+ T, CD8+ T, Treg lymphocytes, as well as total CD56+ NK, CD56^dim^ NK, CD56^bright^ NK, and NKT-like cells were determined and compared based on the flow cytometric gating strategy indicated on Figure 1.

We found that in the E-PBMC samples, a significantly lower number of CD3+ T, CD4+ T, and Treg cells were detected; however, the frequency of total NK and CD56^dim^ NK cells was significantly higher in the affected group when compared to NE samples. As peritoneal fluid was also collected, we could compare the local and peripheral distribution of these subsets in endometriosis. In the E-PTL samples, we detected a significantly higher number of CD8+ T, CD56^bright^ NK, and NKT-like cells than in the periphery. In contrast, the frequency of CD4+ T and Treg cells were significantly lower in the E-PTL samples than in the E-PBMC (Table 2). 

**Figure 2:**
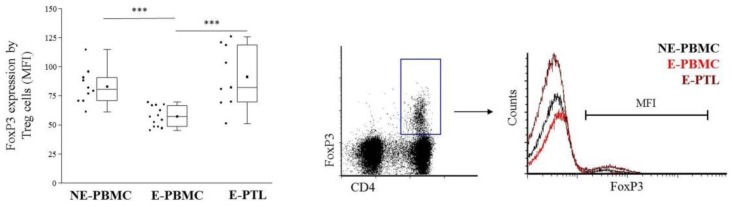
Comparison of the Mean Fluorescent Intensity (MFI) values of FoxP3 positive peripheral (PBMC) and peritoneal (PTL) Treg cells in non-endometriotic (NE) women and patients with endometriosis (E). **Left:** The solid bars represent medians of 10, 12, and 11 determinations, the boxes show the interquartile ranges, and the whiskers show the most extreme observations. The middle square within the box represents the mean value. Statistically significant differences with *p*-values of *** < 0.01 are indicated. **Right:** representative dot plot and histogram figures showing the analysis of CD4+/FoxP3+ Treg cells in NE-PBMC, E-PBMC and E-PTL samples.

It is known that the level of FoxP3 expression by Treg cells is proportional to their suppressive capacity [36]. As literature data indicate that Treg dysfunction plays an important role in the development of endometriosis, we compared their relative FoxP3 expression in NE-PBMC, E-PBMC, and E-PTL cohorts. As indicated in Figure 2, the Mean Fluorescent Intensity (MFI) values of FoxP3 by Treg cells were significantly lower in the E-PBMC samples than that of the NE-PBMC specimens and showed the lowest MFI values when compared to the E-PTL Tregs of the evolved patients. 

### 2.2. Altered TIM-3 Expression by Peripheral Blood and Peritoneal Fluid Mononuclear Cell Subsets in Patients with Endometriosis and Non-Endometriotic Women

Previously, Popovici et al. [28] have found that the mRNA and protein expression of Gal-9 is exclusively increased at the mid- and late-secretory and decidual phases of normal endometrium. Furthermore, our recent study demonstrated a Gal-9 mRNA overexpression in ectopic endometriotic lesions and a significantly increased soluble Gal-9 level in the serum of endometriosis-affected patients compared to non-endometriotic controls [31]. Although these data pose a fundamental role of Gal-9 in endometriosis, the involvement of the TIM-3/Gal-9 regulatory pathway in the development of endometriosis-associated immunological abnormalities is not yet known. Therefore, our next goal was to characterize the cell surface expression of the TIM-3 immune-checkpoint receptor on different PBMC subsets of the NE group and in the E-PBMC and E-PTL samples of the endometriosis cohort. 

**Figure 3:**
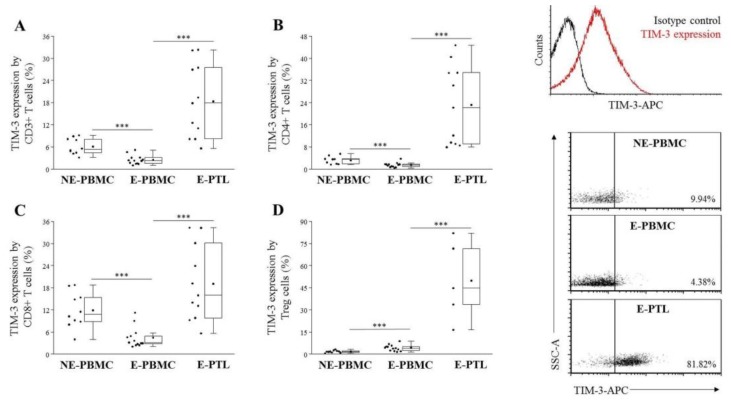
Comparison of the TIM-3 receptor expression by peripheral (PBMC) and peritoneal (PTL) CD3+ T (**A**), CD4+ T (**B**), CD8+ T (**C**) and Treg (**D**) cell subpopulations in non-endometriotic (NE) women and patients with endometriosis (E). **Left:** the solid bars represent medians of 10, 12, and 11 determinations; the boxes show the interquartile ranges, and the whiskers show the most extreme observations. The middle square within the box represents the mean value. Statistically significant differences with *p*-values of *** < 0.01 are indicated. **Right:** representative histograms and dot plots showing TIM-3 receptor expression by T cells in NE-PBMC, E-PBMC and E-PTL samples.

**Figure 4:**
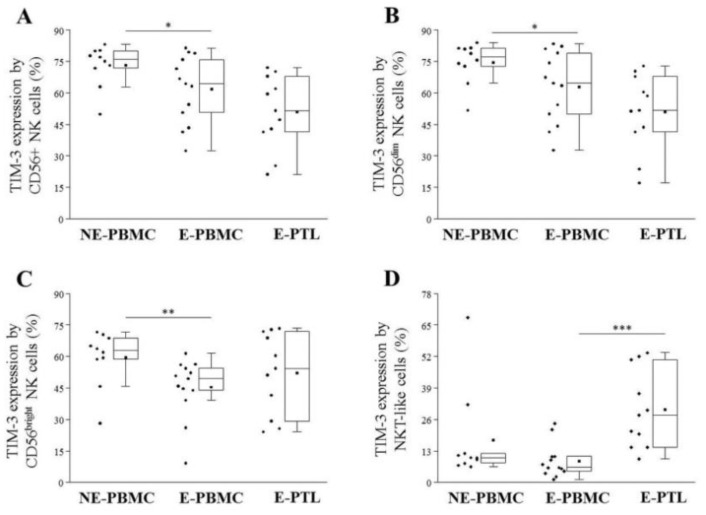
Comparison of the TIM-3 receptor expression by peripheral (PBMC) and peritoneal (PTL) CD56+ NK (**A**), CD56^dim^ NK (**B**), CD56^bright^ (**C**) and NKT-like (**D**) cell subpopulations in non-endometriotic (NE) women and patients with endometriosis (E). The solid bars represent medians of 10, 12, and 11 determinations, the boxes show the interquartile ranges, and the whiskers show the most extreme observations. The middle square within the box shows the mean value. Statistically significant differences for *p*-values of *** < 0.01, ** < 0.03, and * < 0.05 are indicated.

When we compared the TIM-3 expression by different subsets of peripheral T cells, a significantly decreased labeling was observed on CD3+ T, CD4+ T, and CD8+ T cells, however, a significantly elevated positivity was found on FoxP3+ Treg cells of the examined patients when compared them to NE-PBMC group (Figure 3A–D). E-PTL cells exhibited markedly increased TIM-3 labeling on all of the examined T subsets (Figure 3A–D) in comparison to the E-PBMC samples. In contrast, a significantly decreased TIM-3 positivity was observed on all of the tested peripheral CD56+ NK subsets (Figure 4A–C). At the same time, its labeling was markedly elevated on the peritoneal NKT-like cells of women with endometriosis (Figure 4D).

### 2.3. Differential Expression of Cell Surface Galectin-9 by Peripheral Blood and Peritoneal Fluid Mononuclear Cell Subsets in Patients with Endometriosis and Non-Endometriotic Women

**Figure 5:**
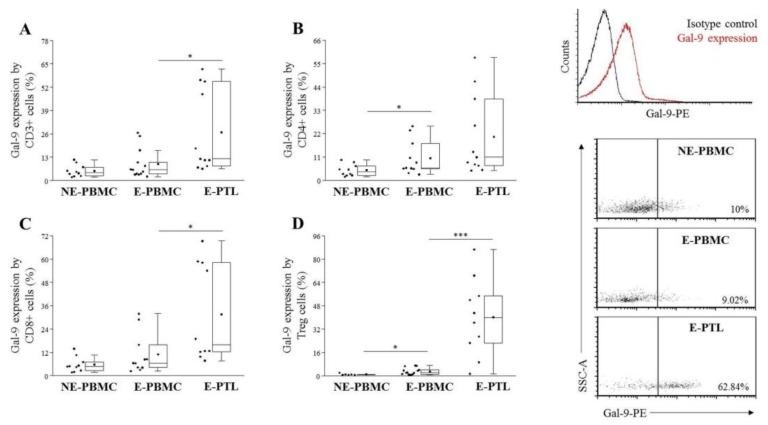
Comparison of the surface Gal-9 ligand expression by peripheral (PBMC) and peritoneal (PTL) CD3+ T (**A**), CD4+ T (**B**), CD8+ T (**C**) and Treg (**D**) cell subpopulations in non-endometriotic (NE) women and patients with endometriosis (E). **Left:** the solid bars represent medians of 10, 12, and 11 determinations; the boxes show the interquartile ranges, and the whiskers show the most extreme observations. The middle square within the box shows the mean value. Statistically significant differences for *p*-values of *** < 0.01 and * < 0.05 are indicated. **Right:** representative histograms and dot plots showing Gal-9 ligand expression by T cells in NE-PBMC, E-PBMC and E-PTL samples.

Although the overexpression of intracellular and secreted Gal-9 was previously proven by our research group in endometriosis [31], the cell surface expression of this lectin has not yet been characterized in this disorder. Comparing the surface Gal-9 positivity on PB-T cells, a significantly elevated expression was revealed on peripheral CD4+ T and Treg cells of E-samples than in the NE- group (Figure 5B,D). In addition, a markedly increased Gal-9 expression was also found on CD3+ T, CD8+ T, and Treg cells isolated from the PF of the affected women when compared to the periphery of the same group (Figure 5A,C,D). 

**Figure 6:**
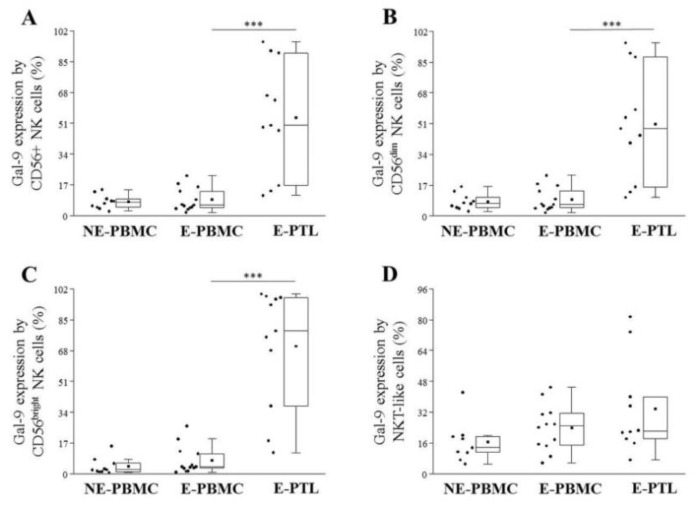
Comparison of the cell surface Gal-9 expression by peripheral (PBMC) and peritoneal (PTL) CD56+ NK (**A**), CD56^dim^ NK (**B**), CD56^bright^ (**C**) and NKT-like (**D**) cell subpopulations in non-endometriotic (NE) women and patients with endometriosis (E). The solid bars represent medians of 10, 12, and 11 determinations; the boxes show the interquartile ranges, and the whiskers show the most extreme observations. The middle square within the box shows the mean value. Statistically significant differences for *p*-values of *** < 0.01 are indicated.

Analyzing the cell surface Gal-9 expression on E-PTL NK cells, a significantly increased labeling was detected on either of the total NK, the CD56^dim^ NK, and CD56^bright^ NK cells in comparison to their peripheral counterparts. The Gal-9 positivity of NKT-like cells was not altered (Figure 6A–D).

## 3. Discussion

Endometriosis is a progressive, chronic, estrogen-dependent inflammatory disorder that is associated with chronic pelvic pain and infertility. Although an in-depth understanding of its pathophysiology is still largely elusive, it is well accepted that dysregulated immune-, vascular- and neuroendocrine pathways are highly involved in the development of this disorder. Studies published to date suggest that endometriosis could be considered a local disease with systemic subclinical manifestations [37]; however, the involvement of immunophenotypic alterations in its pathogenesis is still a subject of controversy. 

In this study, multicolor flow cytometry was used to characterize the distribution and to compare the cell surface expression of TIM-3 and Gal-9 molecules on different peripheral and peritoneal T and NK cell subsets of women with and without endometriosis. This research was subjected to some limitations that have to be considered and could be addressed in future research. The first limitation is related to the restricted sample size that was used for analysis. The second limitation concerns methodical problems. Albeit all endometriosis cases were confirmed by laparoscopy and their clinical data were evaluated in detail (Table 1), but clinical scoring was not done. 

Furthermore, as our controls were non-endometriotic women, their pelvic status was not examined by laparoscopy, and under the EU-GDPR and the Privacy, Informational and Healthy data Act regulations their sensitive personal and clinical data were not disclosed to the research team to fulfill the comparative demographic analysis. Finally, we restricted our trials only on immunophenotypic analysis, but functional tests were not performed as merely frozen cells were available. Despite these limitations, the strength of our present work is to highlight a critical role of the immunological dysregulation in endometriosis and to expose the involvement of the TIM-3/Gal-9 immune checkpoint pathway in this gynecological disorder. 

The phenotypic characterization of different PBMC populations revealed significantly lower frequencies of CD3+ T, CD4+ T, and Treg cells in endometriosis compared to non-endometriotic controls. In addition, a markedly higher number of peripheral CD56^dim^ NK, a slightly elevated CD56^bright^ NK and decreased NKT-like frequencies were also found in the affected cohort. Investigating these lymphocyte subsets in the peritoneal fluid of the affected women showed a significantly lower percentage of CD4+ T, Treg, and CD56^dim^ NK cells, a higher number of CD8+ T, CD56^bright^ NK, and NKT-like cells compared to the periphery. Albeit comparison of the peripheral CD4+/CD8+ and CD4+/Treg ratios did not result in any significant differences between the NE-PBMC and E-PBMC samples, the CD4+/CD8+ ratio was inverted in the PF of the affected women, suggesting a peritoneal redistribution of CD8+ T cells in this disease (Table 2). Confronting the known literature data with our recent findings indicated, that while some of the earlier reports were in accordance with our data [7,38,39,40], others were contrary with our results [41,42,43,44], or did not find any significant difference [10,45] in the distribution of PBMC or PTL subsets in women with or without endometriosis. Based on these inconsistent results, we conceive that not the quantitative differences, but the dysregulated immune response might be the major contributing factor in the immunopathogenesis of this disease. 

As ectopic implants closely interact with the surrounding microenvironment, altered or disturbed expression of molecules that can modulate their survival could be involved in the expansion of the ectopic lesions. One of these molecules might be the β-galactoside-binding galectins, which are pivotal in the regulation of cell adhesion, migration, invasion, angiogenesis, and in the control of innate and adaptive immunity [27,46,47]. Although the roles of Gal-1 and Gal-3 have already been described in endometriosis [48,49], the involvement of Gal-9 in the pathogenesis of this disease is still not known. 

Gal-9 is reported to have distinct functions intracellularly, in the extracellular compartments, or on the cell surface [9]. Recently we have demonstrated that Gal-9 mRNA is overexpressed in the eutopic endometrium, invectopic lesions, and in E-PTL of patients with endometriosis. In addition, we detected increased soluble Gal-9 levels in the serum of the affected patients compared to NE-women, which was positively correlated with the severity of the disease [31]. In the present study, we detected a notable cell surface Gal-9 positivity 3.56 ± 4.76%–70.46 ± 32.98% [mean ± SD%] of the examined T and NK subpopulations and observed that (except NKT-like cells) its presence was more prominent on the E-PTL subsets than in the periphery, which is in contrast to the earlier published data of Madireddi et al. [50] who could not detect Gal-9 expression on the cell surface of activated T cells; however, this group examined mouse lymphocytes instead of human T cells. We established that the surface Gal-9 positivity was significantly elevated on CD4+ T and Treg cells isolated from E-PBMC samples in comparison to NE-PBMC. In addition, we revealed that the Gal-9 expression was significantly elevated by peritoneal CD8+ T, CD56+ NK, CD56^dim^ NK, and CD56^bright^ NK cells compared to their peripheral counterparts showing the highest expression on peritoneal Treg cells. Although the surface Gal-9 positivity of peritoneal CD4+ T cells was also elevated, it did not reach the level of statistical significance. 

The best characterized binding partner of Gal-9 is the TIM-3 immune checkpoint receptor, which was initially identified on terminally differentiated IFN-γ producing helper and cytotoxic T cells. During physiological conditions, engagement of TIM-3 with Gal-9 influences T cell tolerance negatively regulates IFN-γ secretion, and induces apoptosis of Th1 and Th17 cells; hence plays an important role in the regulation of Th1/Th17 polarization. In our previous research immune checkpoint interactions were examined in the context of healthy and pathological pregnancies although much fewer studies have been published about the TIM-3 and Gal-9 molecules in the pathogenesis of endometriosis [51,52]. In the present study, we found a decreased CD4+ T cell number in endometriosis, which was more prominent in the PF than in the PB of the patients. In addition, we found that the TIM-3 receptor expression by CD4+ T cells was significantly lower in the PB than in the PF, resulting in a diminished TIM-3/Gal-9-dependent regulatory impact on the periphery. These results indicate that the “suicidogenic” effect of high dose Gal-9 on Th1 and Th17 cells is more robust in the pelvis, where ectopic lesions are present, and a higher level of soluble lectin is produced. 

In our study, we detected a significantly increased TIM-3 expression by CD8+ T cells in the PF of women with endometriosis compared to the periphery. As activated CD8+ T cells are less susceptible to the death-inducing effect of Gal-9 than CD4+ Th1 cells [53] besides local redistribution, it might explain why we observed a higher number of CD8+ T cell in the PF samples. In addition, we showed that although the percentage of Treg cells was decreased, their surface Gal-9 and TIM-3 expression were significantly elevated in both the PB and, more abundantly, in the PF of patients with endometriosis. Tanaka et al. [36] found a significantly decreased CD45RA-/Foxp3^high^ activated (suppressive) Treg number in the eutopic endometrium and endometrioma samples of the affected women which were in accordance with our recent data Albeit elevated TIM-3 receptor expression identifies a Treg subset highly effective in inhibiting pathological Th1 and Th17-biased immune response [54], we suppose, that the observed decrease in peripheral and peritoneal Treg numbers and the decreased FoxP3 expression may mark diminished regulatory function in endometriosis. In virtue of the above-mentioned data, we reconfirm a theory of dysregulated immune response wherein the decreased suppressive capacity of Treg cells exaggerates peritoneal inflammation, stimulates local angiogenesis, thus facilitates the progression of the disease. 

Previous literature data indicate that Gal-9 promotes the differentiation and FoxP3 expression of Treg cells while suppressing the development of Th17 cells [15]. As earlier, we revealed a significantly elevated Gal-9 level in the serum of women with endometriosis [31], it seems conflicting, why we could not see the elevation of the Treg subpopulation in this disorder? It is well known now that fine regulation of the genetic signature of suppressive activity is critical for the development of Treg cells and the optimal control of the local immune response. In the context of endometriosis, we can imagine that a local inflammatory environment and strong immune-activation might have a disruptive effect on the stability of Tregs and may reprogram them towards immune-boosting or autoreactive effectors even at the presence of high Gal-9 level as indicated by present reports [55,56]. 

In contrast to Tregs, we found that the TIM-3 receptor expression was significantly lower on both CD56^dim^ NK and CD56^bright^ NK subsets of the E-PBMC samples that on that of the NE-PBMC lymphocytes. It was found that the effect of Gal-9 on NK cells is bimodal since, as an activating co-receptor, it can enhance the IFNγ production of TIM-3^high+^ NK cells, but in some circumstances, it can also deliver inhibitory signals during chronic conditions [57,58,59]. We suppose that similarly to chronic HIV infection, persistent signaling through TIM-3 receptor induced by high local Gal-9 production might result in the downregulation of TIM-3 expression by NK cells; therefore, it can contribute to the previously described NK cell dysfunction in endometriosis. In addition and based on the recently published data of Motamedi et al. [60], we propose that the significantly elevated number of Gal-9+ NK cells in the PF of patients with endometriosis may mark functionally impaired killer cells, that express negligible amounts of perforin and granzyme (which is detrimental to their cytotoxic abilities) but can produce a high level of IFNγ, where the role is particularly highlighted in the pathogenesis of endometriosis.

In the current study, we revealed a significantly elevated TIM-3 receptor expression on peritoneal NKT-like cells of endometriosis-affected women in comparison to the periphery. As TIM-3+ NKT-like cells can proliferate and secrete a range of cytokines upon stimulation [61], we think, that they are in a functional activated state. Based on the results of Kadowaki et al. [62], we hypothesize that a high local Gal-9 concentration plays an important role in the regulation of TIM-3+ NKT-like cells by inducing their local, activation-dependent proliferation, and enhancing their capacity to produce a high amount of IL-17. Further studies are required to ascertain how the engagement of TIM-3 by Gal-9 affects the survival and cytokine production on different NKT-like subsets in endometriosis. 

## 4. Materials and Methods

### 4.1. Ethical Approval

The trial was approved by the Regional Committee for Research Ethics of the University of Pecs Medical School, Hungary (2015) and was recorded on the Institutional Research Register with a registration number of 5816. The study protocol conforms to the ethical guidelines of the 1975 Declaration of Helsinki. Written informed consent was obtained from all participating individuals.

### 4.2. Patients and Sample Collection

Specimens were collected at the Department of Obstetrics and Gynecology, Medical School, University of Pecs, Hungary. Diagnostic or operative laparoscopy was performed in a total of 23 reproductive age women (range 24–40 years, demographic data are presented in Table 1), whose endometriosis was classified during laparoscopy according to the anatomical localization of the endometriotic lesions. After surgical intervention, endometriosis was confirmed histologically in all of the involved cases. Before surgery, 10 mL of peripheral venous blood were collected from all of the endometriosis-affected women by venipuncture (*n* = 12) in K2EDTA containing tubes (E-PBMC cohort). In *n* = 11 cases, peritoneal fluid was aspirated during laparoscopy from the pouch of Douglas before any surgical manipulation (E-PTL cohort). Special precaution was taken to avoid blood or other fluid (saline, methylene blue dye) contamination. In addition, peripheral venous blood from age-matched female volunteers (*n* = 10) was obtained from the Hungarian National Blood Transfusion Center, Regional Centre Pecs, Hungary, and were used as non-endometriotic control samples (NE-PBMC cohort). The health status of the blood volunteers was identified by a mini-interview, and all of those women who reported gynecologic problems were excluded from the study. Under the EU-GDPR and due to the Privacy, Informational and Healthy data Act regulations, all personal and health-related data obtained about the donors during blood donation were processed anonymously, confidentially and securely, and were not provided to the Research team for further demographic analysis. After collection, all of the biological specimens were immediately transported to the Lab for further analysis. In the whole study, all of the examined peripheral blood and peritoneal fluid samples were handled and treated uniformly.

### 4.3. Lymphocyte Separation, Cryopreservation, and Thawing

Peripheral blood mononuclear cells of non-endometriotic and endometriosis-affected women were purified from K2EDTA-treated venous blood samples on Ficoll–Paque (GE Healthcare, Little Chalfont, UK), density- gradient centrifugation at 2000 rpm for 20 min. PBMC were collected from the interphase, washed in complete Rosewell Park Memorial Institute medium [RPMI1640 (Lonza Basel, Switzerland)] supplemented with 10% fetal bovine serum (FBS, Gibco by Life Technologies, Grand Island, NY, USA) for 6 min at 1200 rpm. The cell-containing pellet was resuspended in heat-inactivated human AB-serum (Biowest, Nuaillé, France) containing 10% Dimethyl-sulfoxide (DMSO, Sigma-Aldrich St. Louis, MO, USA) for cryoprotection. Next, the isolated PBMC were aliquoted in cryovials and stored at −80 °C in the mechanical freezer. Thawing was carried out on the day of immunolabeling. Aspirated peritoneal fluid samples were centrifuged for 10 min at 3000 rpm, and the obtained cell-pellet was resuspended in complete RPMI medium. Peritoneal fluid leukocytes were isolated on Ficoll-Paque gradient similarly to PBMC, resuspended in human AB serum supplemented with 10% DMSO, next aliquoted in cryovials and stored in −80 °C freezer until further use.

### 4.4. Antibodies

Freshly thawed PBMC and PTL samples were used for surface and intracellular immuno-labeling and flow cytometric analysis. The following antibodies were used: fluorescein isothiocyanate (FITC)-conjugated anti-human CD4 (Clone: RPA-T4, BD Biosciences, Franklin Lakes, NJ, USA), phycoerythrin (PE)-conjugated anti-human Gal-9 (Clone: 9M1–3, Biolegend, San Diego, CA, USA), PE-conjugated anti-human TIM-3 (Clone: 344823, R&D Systems, Minneapolis, MN, USA), Peridinin-chlorophyll protein (PerCP)-conjugated anti-human CD56 (Clone: B159, BD Biosciences Franklin Lakes, NJ, USA), allophycocyanin (APC)-conjugated anti-human TIM-3 (Clone: 344823, R&D Systems, Minneapolis, MN, USA), APC-conjugated anti-human FoxP3 (Clone: 236A/E7, eBioscience, Santa Clara, CA, USA), APC-H7 conjugated anti-human CD8 (Clone: SK1, BD Biosciences, Franklin Lakes, NJ, USA) and Brilliant Violet (BV) 510-conjugated anti-human CD3 (Clone: UCHT1, BD Biosciences, Franklin Lakes, NJ, USA). Control antibodies included isotype-matched FITC-, PE-, APC-, APC-H7, or BV510-conjugated mouse antibodies. 

### 4.5. Immunolabeling of Leukocytes and Flow Cytometric Analysis

Cryopreserved PBMC or PTL cells were thawed in a 37 °C water bath as quickly as possible and was washed twice with RPMI medium to remove DMSO. 1 × 10^6^ cells were resuspended in 100 μL Dulbecco’s phosphate saline buffer/tube (D-PBS, Lonza, Basel, Switzerland) and were incubated for 30 min at room temperature with fluorochrome-conjugated monoclonal antibodies. After staining, the cells were washed with D-PBS, fixed in 300 μL of 1% paraformaldehyde (PFA) solution, and stored at 4 °C in darkness until Fluorescence-activated cell sorting (FACS) analysis. Flow cytometry was carried out using the BD FACSCanto II flow cytometer (BD Immunocytometry Systems, Franklin Lakes, NJ, USA) equipped with BD FACSDIVA V6 software (BD Biosciences, Franklin Lakes, NJ, USA) for data acquisition and analysis.

### 4.6. FoxP3 Intracellular Labeling

After surface labeling, intracellular FoxP3 staining was also performed using the FoxP3 Staining Buffer Set (eBioscience, Santa Clara, CA, USA) according to the manufacturer’s protocol. Briefly, isolated leukocytes were permeabilized in 1 mL Fixation/Permeabilization buffer (Concentrate/Diluent 1:4) at 4 °C for 1 h. Then the samples were washed twice in 1× F/P buffer and stained with APC-conjugated anti-human FoxP3 monoclonal antibody at 4 °C for 1 h in the dark. After 2× washing cells were resuspended in D-PBS, fixed with 1% PFA, and evaluated by FACS. 

### 4.7. Statistical Analysis

Clinical data were provided to the Research Team only after executing the study. The localization of endometriotic lesions was established during laparoscopy, and it was used as a reference for the anatomical classification of endometriosis. During statistical analysis, the obtained FACS data were first evaluated by descriptive statistical methods such as mean, SD, and frequency and the distribution of the data sets was determined. As only two independent groups (NE-PBMC vs. E-PBMC or E-PBMC vs. E-PTL) were compared at once and the examined values demonstrated normal distribution, two-tailed Student’s t-test was used as a statistical method to compare sample means on one variable. Multiple comparisons were not made. Differences were considered significant if the calculated *p*-value was ≤0.05.

## 5. Conclusions

Our results indicate a persistent activation and a disturbed regulatory function of the TIM-3/Gal-9 pathway in endometriosis, suggesting its potential impact in the evolution of the altered immune-effector mechanisms in the survival of ectopic lesions and the development of endometriosis-associated reproductive failures. We hope that our results may add deeper insight into the pathogenesis of this enigmatic disease by revealing a dysfunctional regulatory mechanism that may contribute to the immunological evolvement of endometriosis and may pave the way for the development of new therapeutic possibilities for the clinical practice. 

## Figures and Tables

**Table 1:** Demographic and clinical characteristics of the endometriosis affected cohorts.

	E-PBMC (*n* = 12)	E-PTL (*n* = 11)	*p*-Value
**Mean age (years ± SD)**	31.92 ± 4.337	29.00 ± 4.313	0.1218
**Age ranges at diagnosis (years)**	26–40	24–38	-
**Mean BMI (kg/m^2^ ± SD)**	24.11 ± 4.895	21.09 ± 2.880	0.1009
**Gynecological History – *n* (%)**			
Normal cycle length (25–35 days)	4 (33.33)	3 (27.27)	-
Irregular cycle	4 (33.33)	4 (36.36)	-
Suppressed cycle (GnRH analogue)	0 (0.00)	1 (9.09)	-
**Fertility Data – *n* (%)**			
Previous normal pregnancy	4 (33.33)	0 (0.00)	-
Previous pathological pregnancy	2 (16.67)	1 (9.09)	-
Infertility	7 (58.33)	9 (81.82)	-
**Endometriosis-Related Data – *n* (%)**			
Previous laparoscopic intervention	8 (66.67)	8 (72.73)	-
Pharmacologic treatment	3 (25.00)	4 (36.36)	-
Pelvic pain	8 (66.67)	5 (45.45)	-
Dysmenorrhea	9 (75.00)	9 (81.82)	-
Dyspareunia	5 (41.67)	6 (54.55)	-
Dyschesia	8 (66.67)	8 (72.73)	-
Dysuria	1 (8.33)	1 (9.09)	-
**Type of Endometriosis – *n* (%)**			
Peritoneal	1 (8.33)	3 (27.27)	-
Ovarian endometriosis	2 (16.67)	2 (18.18)	-
Deep infiltrating endometriosis (DIE)	3 (25.00)	2 (18.18)	-
Combined (DIE + other)	6 (50.00)	4 (36.36)	-
**Other Associated Diseases – *n* (%)**			
Autoimmunity	0 (0.00)	1 (9.09)	-
Insulin resistance	1 (8.33)	2 (18.18)	-

Comparative demographic and clinical analysis of data collected from endometriosis-affected cohorts. Statistical analysis was made by Student’s *t*-test for continuous variables and the calculated results were indicated as mean values ± standard deviation [mean ± SD]. In all of the categorical variables, the actual case number and percentage of total cases [*n* (%)] are presented.

**Table 2:** Phenotype characteristics of the analyzed mononuclear cell populations.

	NE-PBMC	E-PBMC	*p*-Value	E-PBMC	E-PTL	*p*-Value
**CD3**	**63.85 ± 8.49**	**53.52 ± 11.43**	**<0.03**	53.52 ± 11.43	57.09 ± 4.10	*n*.s.
**CD4**	**37.09 ± 6.50**	**26.45 ± 8.83**	**<0.01**	**26.45 ± 8.83**	**14.90 ± 4.66**	**<0.001**
**CD8**	20.78 ± 7.27	18.73 ± 3.33	*n*.s.	**18.73 ± 3.33**	**32.11 ± 9.90**	**<0.01**
**Treg**	**3.41 ± 1.20**	**1.56 ± 0.53**	**<0.001**	**1.56 ± 0.53**	**1.03 ± 0.47**	**<0.03**
**CD4/CD8**	2.03 ± 0.84	1.45 ± 0.61	*n*.s.	**2.03 ± 0.84**	**0.49 ± 0.16**	**<0.01**
**CD4/Treg**	11.88 ± 3.86	18.01 ± 5.88	*n*.s.	11.88 ± 3.86	20.10 ± 13.16	*n*.s.
**CD56+ NK**	**15.34 ± 7.20**	**26.40 ± 10.99**	**<0.02**	26.40 ± 10.99	24.07 ± 14.06	*n*.s.
**CD56^dim^ NK**	**14.01 ± 6.75**	**25.10 ± 10.85**	**<0.01**	25.10 ± 10.85	19.03 ± 12.80	*n*.s.
**CD56^bright^ NK**	1.36 ± 0.69	1.60 ± 0.91	*n*.s.	**1.60 ± 0.91**	**5.30 ± 4.09**	**<0.01**
**NKT-like**	8.57 ± 3.58	6.38 ± 4.61	*n*.s.	**6.38 ± 4.61**	**12.72 ± 4.48**	**<0.01**

Statistical comparisons were performed by Student’s *t*-test between peripheral blood mononuclear cells of non-endometriotic women (NE-PBMC) vs. patients with endometriosis (E-PBMC), and between peripheral (E-PBMC) vs peritoneal leukocytes of endometriosis affected women (E-PTL). The calculated results were presented as the mean value ± standard deviation (mean ± SD) and the differences were considered significant when the *p*-values were ≤0.05. The percentage of lymphocyte subpopulations and their *p*-values indicating significant differences are marked in bold and “*n*.s.” marks non-significant values.

## Abbreviations

TIM	T-cell immunoglobulin and mucin domain
Gal	Galectin
PB	Peripheral blood
PF	Peritoneal fluid
NK	Natural Killer cell
CD	Cluster of differentiation
Treg	Regulatory T cell
FoxP3	Forkhead box P3
Th	T helper cell
TCR	T cell receptor
IFNγ	Interferon gamma
IL	Interleukin
IL-1β	Interleukin 1 beta
TNFα	Tumor Necrosis Factor alpha
PBMC	Peripheral blood mononuclear cell
NE	Non-endometriotic
E	Endometriosis
PTL	Peritoneal fluid leukocytes
rASRM	revised American Society of Reproductive Medicine
EU	European Union
GDPR	General Data Protection Regulation
SD	Standard deviation
BMI	Body mass index
GnRH	Gonadotropin releasing hormone
DIE	Deep infiltrative endometriosis
NKT	Natural Killer T cell
n.s.	Non-significant
MFI	Mean Fluorescent Intensity
K2EDTA	Dipotassium-Ethylene Diamine Tetraacetic Acid
RPMI	Roswell Park Memorial Institute
FBS	Fetal bovine serum
DMSO	Dimethyl sulfoxide
FITC	Fluorescein isothiocyanate
PE	Phycoerythrin
PerCP	Peridinin-chlorophyll protein
APC	Allophycocyanin
D-PBS	Dulbecco’s phosphate saline buffer
PFA	Paraformaldehyde
FACS	Fluorescence-activated cell sorting
